# Reviewing the characteristics of BRCA and PALB2-related cancers in the precision medicine era

**DOI:** 10.1590/1678-4685-GMB-2018-0104

**Published:** 2019-04-29

**Authors:** Gabriel S. Macedo, Barbara Alemar, Patricia Ashton-Prolla

**Affiliations:** 1 Post-Graduate Program in Genetics and Molecular Biology, Universidade Federal do Rio Grande do Sul (UFRGS), Porto Alegre, RS, Brazil; 2 Precision Medicine Program, Hospital de Clínicas de Porto Alegre, Porto Alegre, RS, Brazil

**Keywords:** BRCA1, BRCA2, homologous recombination, cancer predisposition, PARP inhibitors

## Abstract

Germline mutations in *BRCA1* and *BRCA2* (BRCA) genes confer high risk of developing cancer, especially breast and ovarian tumors. Since the cloning of these tumor suppressor genes over two decades ago, a significant amount of research has been done. Most recently*,* monoallelic loss-of-function mutations in *PALB2 have also been shown to increase the risk of breast cancer. The identification of BRCA1, BRCA2 and PALB2 as proteins involved in DNA double-strand break repair by homologous recombination and of the impact of complete loss of BRCA1 or BRCA2 within tumors have allowed the development of novel therapeutic approaches for patients with germline or somatic mutations in said genes. Despite the advances, especially in the clinical use of PARP inhibitors, key gaps remain. Now, new roles for BRCA1 and BRCA2 are emerging and old concepts, such as the classical two-hit hypothesis for tumor suppression, have been questioned, at least for some BRCA functions. Here aspects regarding cancer predisposition, cellular functions, histological and genomic findings in BRCA* and *PALB2*-related tumors will be presented, in addition to an up-to-date review of the evolution and challenges in the development and clinical use of PARP inhibitors.

## *BRCA1, BRCA2* and *PALB2* gene*s*: mutations and associated phenotypes

Hereditary breast and ovarian cancer (HBOC) syndrome is a highly penetrant autosomal dominant disorder accounting for 5-7% of breast cancers (BCs) and 8-13% of epithelial ovarian cancers (EOCs). It is caused mainly by germline mutations in *BRCA1* and/or *BRCA2* (collectively “BRCA” hereafter) (Liu *et al.*, 2012; Roy,*et al.,* 2012; Dai *et al.*, 2018). In *BRCA1* mutation carriers, the average cumulative risks of breast and ovarian tumors by the age of 70 years is 65% and 39%, respectively, whereas in *BRCA2* mutation carriers the corresponding estimates are 45% and 11% (Antoniou *et al.*, 2003). By the age of 80, the cumulative risks of breast and ovarian cancer increase, respectively, to 72% and 44% for *BRCA1* carriers, and 69% and 17% for *BRCA2* carriers (Kuchenbaecker *et al.*, 2017). Additionally, women who carry *BRCA1* germline mutations also have an increased risk of developing fallopian tube and peritoneal cancers (Brose *et al.*, 2002; Finch *et al.*, 2006). Carriers of BRCA1 or BRCA2 mutations may also be in risk for prostate and pancreatic cancer (Levy-Lahad and Friedman, 2007; Consortium, 1999; Thompson et al., 2002; Ferrone et al., 2009). Recently*,* monoallelic loss-of-function mutations in *PALB2* (Partner and Localizer of *BRCA2*) were found to confer predisposition to cancer, with a mean risk of BC in females of 35% by age 70 (Rahman *et al.*, 2007; Antoniou *et al.*, 2014;). Couch *et al.* (2017) showed that pathogenic mutations in *PALB2* are in fact associated with a high-risk of BC (odds ratio 7.5). Based on data from different populations, *PALB2* germline mutations appear to account for approximately 0.7-1.1% of all familial aggregation of BC (Rahman *et al.*, 2007; Buys *et al.*, 2017; Eliade *et al.*, 2017). *PALB2* has also been reported as a susceptibility gene for pancreatic cancer (Jones *et al.*, 2009b; Tischkowitz *et al.*, 2009; Slater *et al.*, 2010).

Germline *BRCA1, BRCA2* and *PALB2* mutations are also associated with an increased risk of developing male breast cancer (MBC) (Thompson *et al.*, 2002; Levy-Lahad and Friedman, 2007; Rahman *et al.*, 2007). Although corresponding to less than 1% of all BC cases, a significant proportion of MBCs arise in a setting of familial BC (Anderson and Badzioch, 1992; Hemminki and Vaittinen, 1999; Weiss *et al.*, 2005). Pathogenic germline mutations in *BRCA2* and *PALB2* have been found in 5-40% (Thorlacius *et al.*, 1997; Basham *et al.*, 2002; Ding *et al.*, 2011) and 1-2% (Ding *et al.*, 2011) of all MBCs, respectively. However, the association between *BRCA1* germline mutations and MBC is not well established, although several studies have demonstrated that the *BRCA1* germline mutations may contribute to a small fraction of MBC cases (Csokay *et al.*, 1999; Sverdlov *et al.*, 2000; Ottini *et al.*, 2009). It was also reported *BRCA* germline mutations in 28% of men with BC, of which a substantial proportion (8 of 22) occurred in *BRCA1* (Frank *et al.*, 2002).

Different from most HBOC cases, in which monoallelic germline mutations are associated to increased adult-onset predisposition to several tumors, biallelic germline loss-of-function mutations in a set of DNA repair genes, including *BRCA1, BRCA2* and *PALB2,* are associated to a distinct phenotype, characterizing subgroups of Fanconi Anemia (FA) (Howlett *et al.*, 2002; Reid *et al.*, 2007; Sawyer *et al.*, 2015). FA is a rare recessive genetically heterogeneous chromosomal instability disorder characterized by congenital and developmental abnormalities and a high predisposition to cancers (Tischkowitz and Hodgson, 2003). FA is divided into several complementation groups according to the mutated gene (Mathew, 2006). Biallelic mutations in *BRCA2* (also known as *FANCD1*) are identified in around 3-5% of FA cases and are associated with a high risk of aggressive embryonal tumors in early childhood stages (mostly medulloblastomas and nephroblastomas) and/or acute leukemia (Reid *et al.*, 2005; Meyer *et al.*, 2014). The cumulative probability of any tumor in these patients was found to be of 97% by age 5.2 years (Alter *et al.*, 2007). Biallelic *PALB2* (also referred as *FANCN*) pathogenic mutations were identified in families affected with FA and childhood cancer, characterizing a new subtype of the disease (Reid *et al.*, 2007). More recently, biallelic *BRCA1* mutations have also been shown to cause a FA-like phenotype (Sawyer *et al.*, 2015; Freire *et al.*, 2018). It has been proposed that patients with two nonsense mutations may survive as the result of naturally occurring alternative splicing that yields a short but partially functional BRCA1 protein (Seo *et al.*, 2018).

## *BRCA1, BRCA2* and *PALB2* mutations

Located on the long arm of chromosome 17 at 17q21 (Miki *et al.*, 1994), the *BRCA1* tumor suppressor gene is composed by 23 exons encoding for a protein of 1863 amino acids (Connor *et al.*, 1997; Teng *et al.*, 2008). *BRCA2* maps to chromosome 13 (13q12.3) (Connor *et al.*, 1997) and consists of 27 exons coding for 3418 amino acids (Tavtigian *et al.*, 1996). The largest exons in BRCA1 and BRCA2 are exons 10 and 11, respectively, which harbors the majority of mutations identified in patients, most of which are frameshift mutations resulting in missing or nonfunctional proteins (Al-Mulla *et al.*, 2009).

The overall population prevalence of *BRCA1* and *BRCA2* mutation carriers is estimated to be 1 in 400 to 1 in 800, respectively, but varies considerably according to the ethnic group (Ford *et al.*, 1995; Whittemore *et al.*, 1997). For instance, in the Ashkenazi Jewish population two common mutations in *BRCA1* (c.68_69delAG, formerly known as 185delAG, and c.5266dupC, also known as 5382insC) and one common mutation in *BRCA2* (c.5946delT, formerly known as 6174delT) are highly prevalent (approximately 2%) (Struewing *et al.*, 1997; Gabai-Kapara *et al.*, 2014). The most common types of deleterious mutations found in *BRCA1* and *BRCA2* are small frameshift deletions or insertions, nonsense, and splice site mutations (Borg *et al.*, 2010). Interestingly, the genomic regions of both *BRCA1* and *BRCA2* genes are composed by a very high density of repetitive DNA elements, comprising approximately 47% of *BRCA1* (42% Alu sequences and 5% non-Alu repeats) and *BRCA2* (20% Alu and 27% LINE and MER repetitive DNA) sequence (Welcsh and King, 2001). Given these characteristics, it is not surprising that Alu-mediated genomic rearrangements within both genes have been observed (Qian *et al.*, 2017). Nevertheless, large rearrangements have been estimated to occur in 0-40% of carriers, depending of the population, and should always be investigated when initial sequencing analysis not sensitive for their detection are reported as negative (Ewald *et al.*, 2009). More recently, due to the possibility of identification of compound heterozygotes, genetic testing guidelines have recommended sequencing and gene rearrangement testing in all suspected cases (NCCN, 2017).

A large number of rare germline variants has been reported throughout both genes according to the Breast Cancer Information Core website (BIC) (approximately 1800 mutations in *BRCA*1 and 2000 mutations in *BRCA*2), and the majority of those have not been reported as recurrent (Breast Cancer Information Core; http://www.research.nhgri.nih.gov/bic). Moreover, around 15% of individuals without any clear pathogenic variant in the *BRCA1* or *BRCA2* genes and about 5-7% of all individuals who undergo *BRCA1* and *BRCA2* testing will be found to have a variant of uncertain significance (VUS), which include missense changes, small in-frame deletions or insertions, as well as alterations in non-coding or in untranslated regions (Plon *et al.*, 2008; Ready *et al.*, 2011; Alemar *et al.*, 2017). Identification of VUS has become a huge challenge when tailoring genetic counseling and disease prevention strategies related to HBOC syndrome (Cheon *et al.*, 2014). Some criteria, such as functional assays, have been proposed to ascertain the pathogenicity of *BRCA1/BRCA2* VUS (Toland and Andreassen, 2017).

The spectrum of *PALB2* mutations is similar to that found in *BRCA1* and *BRCA2* genes, in which protein truncating mutations are distributed throughout the coding regions. However, in contrast to its partners, there is only a small number of pathogenic (or likely pathogenic) missense mutations in the gene, being the vast majority frameshift and nonsense mutations (Southey *et al.*, 2013). Interestingly, in the Finnish population only one mutation in *PALB2* was described (c.1592delT). This founder mutation occurs in 0.2% of the general population and is associated with a 6-fold increased risk of BC (Erkko *et al.*, 2007, 2008; Haanpää *et al.*, 2013).

## Biological functions and impact of mutations

### BRCA1, BRCA2 and PALB2 functions

Few years after the discovery of *BRCA1* and *BRCA2* genes, many studies were able to show aspects regarding the physical and functional interactions made by BRCA proteins in several biological processes, especially in DNA damage response and maintenance of the chromosomal stability (Venkitaraman, 2001; Nielsen *et al.*, 2016). Although BRCA1 and BRCA2 have clearly different biochemical functions, the precise mechanisms by which these proteins protect chromosome integrity are not completely understood. The differences in terms of intracellular localization during the cell cycle, the complexity of partners that have been reported to interact with BRCA proteins, and the dynamic nature of these properties according to cellular signals suggest that BRCA1 and BRCA2 belong to a subset of proteins that work as “hubs” (Venkitaraman, 2014). More recently, the functional interaction of PALB2 and BRCA proteins as well as their role in DNA damage response has been partially described (Xia *et al.*, 2006; Sy *et al.*, 2009).

The protein products of *BRCA1* and *BRCA2* have been recognized as crucial for effective DNA repair of double-strand breaks (DSB) (Moynahan *et al.*, 1999, 2001). DSB is one of the most cytotoxic types of DNA damage and it may trigger genome rearrangements and cell death (Stracker *et al.*, 2013). DSB repair is mainly undertaken by homologous recombination (HR) and nonhomologous end-joining (NHEJ), two DNA repair pathways that are differentially regulated depending on the phase of the cell cycle and nature of the damage (Burma *et al.*, 2006; Sonoda *et al.*, 2006; Mao *et al.*, 2008). HR, a vital DNA repair pathway that uses the undamaged sister chromatid to repair replication-associated DSBs, is a commonly error free pathway especially important during the S and G2 phases of the cell cycle. HR involves proteins that can detect broken ends (sensors, e.g ATM/ATR), repair the damage (effectors, e.g BRCA2 and RAD51) and connect both (mediators, e.g CHK2 and BRCA1) (Roy *et al.*, 2012). PALB2 is immediately downstream of BRCA1, being required for efficient DNA repair by HR (Zhang *et al.*, 2009). PALB2 absence prevents recruitment of BRCA2 and RAD51 to the DSB site (Xia *et al.*, 2006; Sy *et al.*, 2009).

In addition to HR, the NHEJ DNA repair pathway may be activated as an alternative mechanism of DSB repair (Brandsma and Gent, 2012). NHEJ is active throughout the cell cycle (favored in G1) and promotes direct ligation of the DSB ends, but in an error-prone manner, frequently resulting in small insertions, deletions and translocations (Lieber, 2010). Although there are conflicting results concerning the role of BRCA1 in NHEJ, this DNA repair pathway has been reported to be unaffected in a BRCA1-deficiency context (Baldeyron *et al.*, 2002; Gudmundsdottir and Ashworth 2006). This may be due, at least in part, to the differential involvement of this protein in the NHEJ subpathways. Some studies support the promoting role of BRCA1 in precise NHEJ, while others show a negative regulation (Wang *et al.*, 2006). So far, it seems that BRCA2 and PALB2 are not required for NHEJ DNA repair (Xia *et al.*, 2001; Metzger *et al.*, 2013).

It is remarkable that BRCA1, BRCA2 and PALB2-deficient cells exhibit spontaneously single sister chromatid breaks, quadri and triradial chromosomes, as well as translocations, large deletions, and fusions involving non-homologous chromosomes (Shen *et al.*, 1998; Yu *et al.*, 2000; Moynahan 2002; Nikkilä *et al.*, 2013). Most importantly, DSB seems to be the typical structural aberration found in BRCA-deficient cells, suggesting that HR is important for tumor suppression (Venkitaraman, 2014). Thus, cells that lack BRCA1, BRCA2 or PALB2 repair the lesions by an error-prone mechanism, such as NHEJ (Tutt *et al.*, 2005; Obermeier *et al.*, 2015;). This shift is in agreement with aneuploid features and frequently compromised chromosome segregations found in these cells (Venkitaraman, 2014). Taken together, this data supports the current knowledge that BRCA and PALB2 proteins play important roles in the maintenance of genomic stability, while deficiency of these proteins promotes chromosomal instability and carcinogenesis.

More recently, based on the broad variability of abnormalities found in BRCA knockout and mutated cells, several new functions for *BRCA1* and *BRCA2* genes have emerged. BRCA1 has been implicated in the mitotic spindle-pole assembly, via BRCA1/BARD1 complex. The potent ubiquitin E3 ligase activity of this interaction seems to be fundamental for TPX2 accumulation, a major spindle organizer. This previously unrecognized function likely contributes to its chromosome stability control and tumor suppression (Joukov *et al.*, 2006). Inactivation of BRCA2 also leads to spindle assembly defects and aneuploidies, suggesting a role of BRCA2 in the spindle assembly checkpoint and kinetochore stability (Choi *et al.*, 2012). Moreover, BRCA2 also seems to protect the length of the nascent strand of DNA from degradation at stalled replication forks, since BRCA2-deficient hamster cells show that newly synthesized DNA strands are substantially shorter compared to wild-type BRCA2 cells (Schlacher *et al.*, 2011). Several other studies have also suggested a role for BRCA proteins in chromatin remodeling (Ye *et al.*, 2001), gene expression (Hill *et al.*, 2014), telomere protection (French *et al.*, 2006; Badie *et al.*, 2010), and heterochromatin maintenance (Zhu *et al.*, 2011). However, whether these emerging BRCA functions are required for tumor suppression is unknown.

## The two-hit model of carcinogenesis

Over 40 years ago, Alfred Knudson proposed a model of carcinogenesis in which biallelic mutations in a tumor suppressor gene are required for tumor development (also called Knudson’s “Two Hit” Hypothesis) (Knudson, 1971). Although this has been accepted for many years, recently published data have shown that inactivation of both alleles may not be a rate-limiting step for some tumor suppressor genes (Berger *et al.*, 2011). Haploinsufficiency is one of the mechanisms that may explain phenotypes arising in tumors or normal cells heterozygous for such mutations. This phenomenon, characterized by reduction in the gene dosage as a result of a monoallelic mutation, leads to changes of cellular processes that may contribute to tumorigenesis (Santarosa and Ashworth, 2004). In agreement with the Knudson hypothesis, seminal studies in mice models showed that complete BRCA1, BRCA2 and PALB2 deficiency results in early embryonic lethality. Interestingly, BRCA1, BRCA2 and PALB2 heterozygous mice could not be distinguished from wild-type animals, corroborating the classic recessive model for tumor suppression, at least in animal models (Gowen *et al.*, 1996; Sharan *et al.*, 1997; Rantakari *et al.*, 2010).

In contrast to what has been observed in mice, humans heterozygous for pathogenic *BRCA1, BRCA2* and *PALB2* germline mutations are predisposed to several tumors (Antoniou *et al.*, 2003, 2014; Liu *et al.*, 2012; Roy *et al.*, 2012), and biallelic mutations in these genes result in FA (Howlett *et al.*, 2002; Reid *et al.*, 2007; Sawyer *et al.*, 2015). Although *BRCA1, BRCA2* and *PALB2* have been considered *bona fide* tumor suppressor genes, whose complete loss-of-function due to deletion, mutation, or gene promoter methylation of the wild-type allele is required for carcinogenesis (Narod and Foulkes 2004; Ashworth *et al.*, 2011; Bowman-Colin *et al.*, 2013;), new evidence has challenged this notion and demonstrated that heterozygote mutations in these genes may be sufficient to impact on biological functions. This affects DNA repair and genomic stability function, enabling the development of tumors in humans (Pathania *et al.*, 2014; Sedic *et al.*, 2015). It is still unclear whether inactivation of the wild-type allele is essential for tumor initiation or if that occurs stochastically.

Several studies have shown that although loss of the wild-type allele (loss of heterozygosity, LOH) is common in breast tumors from carriers of germline *BRCA1* or *BRCA2* mutations (BRCA-BCs), not all breast tumors display this feature, suggesting that at least a subset of the BRCA*-*BCs can develop in the absence of BRCA LOH (Osorio *et al.*, 2002; Palacios *et al.*, 2003; Tung *et al.*, 2010; Stefansson *et al.*, 2011; Martins *et al.*, 2012). Indeed, Maxwell *et al.* (2017) evaluated 160 *BRCA* germline mutated breast and ovarian tumors and found that while BRCA1-germline mutant breast and ovarian tumors had LOH in 90% and 93% of all BRCA1-related cases, respectively, BRCA2-germline mutant tumors retained the wild-type allele in 16% of all BRCA2-related ovarian and 46% of BRCA2 breast tumors. On the other hand, conflicting data for PALB2-BCs has been reported. Most studies have focused on the presence of *PALB2* deletions, however, whether the wild-type *PALB2* allele may be silenced through the presence of mutations, somatic rearrangements, or epigenetic events is still unknown (Tsuda *et al.*, 1995; Erkko *et al.*, 2007; Tischkowitz *et al.*, 2007; García *et al.*, 2009; Casadei *et al.*, 2011; Hartley *et al.*, 2014). Although the reason for disparities between mice and humans is still unknown, the short lifespan, low rate of LOH and tissue-specific haploinsufficiency observed in mice may explain these differences (Drost and Jonkers, 2009).

As previously mentioned, haploinsufficiency of BRCA1, BRCA2 and PALB2 genes may be associated to several cellular phenotypes (Buchholz *et al.*, 2002; Lim *et al.*, 2009; Nikkilä *et al.*, 2013). Some data indicate that normal mammary epithelial cells (MEC) from heterozygous for *BRCA* mutations show increased ability for clonal growth, altered differentiation properties, and aberrant expression profiles (Burga *et al.*, 2009; Lim *et al.*, 2009; Bellacosa *et al.*, 2010; Proia *et al.*, 2011; Feilotter *et al.*, 2014). Moreover, supporting this “haploinsufficiency phenotype”, King *et al.* (2007) identified partial or complete LOH involving the mutant rather than wild-type allele in normal epithelium from *BRCA1* and *BRCA2* mutation carriers, possibly due to higher susceptibility to mitotic recombination within these cells. In another study, a comprehensive analysis using wild-type *vs.* heterozygous mutant *BRCA1* MECs and fibroblasts has provided clues regarding the biological mechanisms of haploinsufficiency (Pathania *et al.*, 2014). They demonstrated that all heterozygous mutant *BRCA1* cells exhibited multiple normal *BRCA1* functions, including maintenance of homologous recombination-type double-strand break repair, checkpoint functions, centrosome number control and spindle pole formation. However, these cells exhibited innate haploinsufficiency in their ability to support stalled fork repair and prevent replication stress. In contrast, Martins *et al.* (2012) have identified centrosome abnormalities in the normal breast tissue from *BRCA1* mutations carriers. Moreover, Konishi *et al.* (2011) demonstrated *in vitro* and *in vivo* that heterozygous *BRCA1* mutations confers impaired homology-mediated DNA repair and hypersensitivity to genotoxic stress in MECs. Additional results also revealed higher gene copy number losses and genomic instability in these cells when compared with their respective controls. Taken together, these findings suggest that haploinsufficiency of BRCA1 may accelerate carcinogenesis by facilitating additional genetic alterations. Recently, Savage *et al.,* showed that transcription of the *CYP1A* gene, which encodes an estrogen-metabolizing enzyme, is upregulated in *BRCA1* heterozygous cells. In addition, it was demonstrated that estrogen and estrogen metabolites result in increased DNA DSBs in *BRCA1* heterozygous cells. Altogether, these data suggest that BRCA1 haploinsufficiency could result in DNA damage in tissues under estrogen stimulation and provides some clues regarding why breast and ovarian tissues are mostly affected in BRCA mutation carriers (Savage *et al.,* 2014).

In contrast to *BRCA1*, much less is known about biological mechanisms associated with *BRCA2* and *PALB2* monoallelic mutations. Arnold *et al.* (2006) using lymphoblastoid cell lines, have found lower amounts of the full-length BRCA2 protein in *BRCA2* heterozygote cells compared to *BRCA2* wild-type. This dosage effect of BRCA2 protein was correlated with an increase in DNA DSBs and an impaired repair of these lesions (Arnold *et al.*, 2006). For some mutations (*e.g.*, truncating mutations) lower amounts of BRCA2 protein also lead to increased chromosomal rearrangements and higher rates of sister chromatid exchanges, indicating a higher susceptibility of *BRCA2* heterozygous cells to chromosomal abnormalities (Savelyeva *et al.*, 2001; Kim *et al.*, 2004). Defects in the recruitment of RAD51 to DSB sites and in activating HR have also been reported in BRCA2-deficient cells (Yuan *et al.*, 1999). In a study published by Nikkilä *et al.* (2013), low levels of PALB2 protein, aberrant DNA replication/damage response, as well as elevated chromosome instability was observed in the *PALB2* heterozygote state. Moreover, it has been demonstrated that PALB2 mutation increases error-prone DSB repair, but do not affect HR and RAD51 filament assembly. (Obermeier *et al.*, 2015).

In conclusion, heterozygosity for *BRCA1, BRCA2* and *PALB2* mutations may impair different biological mechanisms. Although the impact of these alterations on carcinogenesis remains unknown, these detectable effects of “one hit” potentially represent early molecular changes in tumorigenesis. However, these findings remain inconclusive since most of the studies done so far used small numbers of samples and non-isogenic cell lines.

## Tumor phenotype and genomic landscape of BRCA1, BRCA2 and PALB2-associated tumors

### Histology and immunophenotype

Invasive ductal carcinoma is the most common histological breast tumor type observed in *BRCA1* and *BRCA2* carriers (Honrado *et al.*, 2005). Other histological subtypes, including medullary and tubular carcinoma, are also found in this subgroup of patients (Mavaddat *et al.*, 2012). A more detailed examination of morphologic features of the tumors has shown that when compared to sporadic BCs, BRCA1 tumors exhibited higher mitotic counts, more lymphocytic infiltration and greater proportion of the tumor with a continuous pushing margin. On the other hand, BRCA2 tumors are less homogeneous, but exhibit a higher score for tubule formation, higher proportion of the tumor perimeter with a continuous pushing margin, and a lower mitotic count than sporadic BCs (Lakhani *et al.*, 1998). The vast majority of BRCA1 tumors are poorly differential (grade 3), while BRCA2 tumors are usually moderately (grade 2) or poorly (grade 3) differentiated (Agnarsson *et al.*, 1998; Lynch *et al.*, 1998; Palacios *et al.*, 2003). These and other findings have suggested that breast tumors arising in *BRCA1* mutation carriers are associated with more aggressive tumor characteristics compared to *BRCA2* mutation carriers (Krammer *et al.*, 2017).

Despite being driven by germline mutations in functionally related genes, *BRCA1*, *BRCA2,* and *PALB2* mutated breast cancers constitute a heterogeneous group of tumors at the immunohistochemical and molecular level (Table 1). In a way akin to the morphological findings, at least 70% of the tumors arising in *BRCA1* mutation carriers display a triple-negative phenotype (estrogen receptor (ER)-negative, progesterone receptor (PR)-negative and human epidermal growth factor 2 (HER2)-negative), and are classified as basal-like molecular subtype according to immunohistochemical and microarray data (Sorlie *et al.*, 2003: Badve *et al.*, 2011; Mavaddat *et al.*, 2012). In contrast, BRCA2 tumors have been classified predominantly as hormone receptor-positive (Melchor *et al.*, 2008; Mavaddat *et al.*, 2012). A significant proportion of these tumors are of unclassified subtype, with intermediate characteristics between Luminal A and B subtypes (Melchor *et al.*, 2008). Furthermore, several reports have shown similar prevalence of ER- and PR-positive disease in *BRCA2* carriers compared with sporadic controls (Armes *et al.*, 1999; Palacios *et al.*, 2005). Regarding PALB2 tumors, a study conducted by Heikkinen *et al.* (2009) found that breast tumors arising in patients carrying a Finnish founder mutation in *PALB2* (c.1592delT) are more likely to have triple-negative phenotype when compared to non-*PALB2* mutation-associated BCs. Additionally, these tumors were more often of higher grade, had greater expression of Ki-67 and were associated to reduced survival (Heikkinen *et al.*, 2009). In the majority of cases, however, the clinical phenotype of PALB2-BC resembles that of BRCA2-BC, since both are predominantly ER- and PR-positive (Bane *et al.*, 2007; Tischkowitz *et al.*, 2007; Teo *et al.*, 2013; Antoniou *et al.*, 2014; Cybulski *et al.*, 2015; Nguyen-Dumont *et al.*, 2015). Furthermore, minimal sclerosis was identified as a predictor of germline *PALB2* mutation status, distinguishing *PALB2* mutation carriers from *BRCA1* and *BRCA2* mutation carriers (Teo *et al.*, 2013).

**Table 1:** Pathological and molecular characteristics of *BRCA1*, *BRCA2* and *PALB2*-associated breast tumors.

	BRCA1 tumors	BRCA2 tumors	PALB2 tumors	References
**Immunophenotype**				
ER-positive	22%	77%	53%	Mavaddat *et al.,* 2012
				Heikkinen *et al.,* 2009
PR-positive	21%	64%	43%	Mavaddat *et al.,* 2012
				Heikkinen *et al.,* 2009
HER2-positive	10%	13%	4%	Mavaddat *et al.,* 2012
				Heikkinen *et al.,* 2009
Cyclin D1	Usually negative	Usually positive	Usually negative/Low	Palacios *et al.,* 2005, Heikkinen *et al.,* 2009
				Armes *et al.,* 1999
Cyclins E, A and B1	Usually positive	Usually negative	-	Palacios *et al.,* 2005
p16, p27 and p21	Usually negative	Usually positive	-	Palacios *et al.,* 2005
PTEN loss	> 80%	-	-	Phuah *et al.,* 2012
				Saal *et al.,* 2008
Basal markers	Usually positive	Usually negative	Usually negative	Honrado *et al.,* 2006, Heikkinen *et al.,* 2009 Armes *et al.,* 1999
Ki-67	Higher expression^a^	Similara	Higher expressiona	Heikkinen *et al.,* 2009
**Genetic alterations**				
*TP53* somatic mutation^b^	67-95%	66%	-	Manié *et al.,* 2009; Crook *et al.,* 1998
*BRCA* or *PALB2* LOH	84-100%	54-83%	0-33%	Martins *et al.,* 2012; Tung *et al.,* 2010; Osorio *et al.,* 2002; Hartley, 2014; Tischkowitz *et al.,* 2007; Maxwell *et al.,* 2017.
*MYC* amplification	18-53%	62%		Network, 2012; Palacios *et al.,* 2003
*CCND1* amplification	0-22%	13-60%		Vaziri *et al.,* 2001; Plevova *et al.*, 2010; Brown *et al.,* 2010;

In addition to a triple-negative phenotype and expression of basal markers, BRCA1 tumors are characterized by high proliferation rate (Foulkes *et al.*, 2003; Lakhani *et al.*, 2005). Overexpression of proteins associated to cell cycle progression (cyclin E, A and B1) as well as low expression of cyclin D1 and cyclin-CDK complex inhibitors such as p16, p27, and p21 has also been observed (Chappuis *et al.*, 2005; Palacios *et al.*, 2005; Honrado *et al.*, 2006). Unlike BRCA1 tumors, BRCA2 tumors seem to be characterized by higher expression of cell cycle proteins, including cyclin D1, cyclin D3, p27, p16, p21, CDK4, CDK2 and CDK1 (Palacios *et al.*, 2005). A recent study found that BRCA tumors are usually positive for PARP1 (non-cleaved), possibly stimulated by DNA breaks and *BRCA* deficiency. Lower expression of RAD51 and BARD1, two key components of DNA damage repair by HR, were also found in BRCA1 and BRCA1/BRCA2 tumors, respectively, when compared with sporadic BCs (Aleskandarany *et al.*, 2015). PALB2 BCs are not different from other breast tumors regarding cytokeratin 5/6 and 17 expression, but show higher expression of Ki-67 and lower cyclin D1 than other familial and sporadic BCs (Heikkinen *et al.*, 2009).

## Link between BRCA1 and ER status

Despite the evident association between BRCA1 tumors and a triple-negative phenotype, the complete mechanisms underlying this correlation are still unclear. Findings of *in vitro* studies have suggested that BRCA1 directly modulates ER expression in BC, and that BRCA1 deficiency would result in an ER-negative phenotype (Hosey *et al.*, 2007; Gorski *et al.*, 2009;). Furthermore, there is evidence showing that the differentiation status of breast stem cells may be regulated by BRCA1 and that these breast tumors originate from ER-negative luminal progenitor cells (Lim *et al.*, 2009; Molyneux *et al.*, 2010). However, at least 20% of all breast tumors arising in the *BRCA1* germline mutation carriers express ER (Mavaddat *et al.*, 2012). Some authors argue that these cancers are not linked to *BRCA1* germline mutations, but most likely constitute sporadic ER-positive tumors (Tung *et al.*, 2010). In contrast, Natrajan *et al.* (2012) using whole genome massively parallel sequencing, showed that ER-positive and ER-negative *BRCA1* cancers share a very similar genomic landscape, therefore suggesting that at least a subset of ER-positive *BRCA1* mutant tumors are not sporadic, but associated with BRCA1 deficiency. In agreement, there are data suggesting that the prevalence of loss of wild-type *BRCA1* between ER+ and ER- invasive BRCA1 breast tumors does not differ (Natrajan *et al.*, 2012). Moreover, it seems that absence of *BRCA1* is not sufficient for breast tumors to harbor an ER-negative phenotype (Joosse, 2012).

## Genomic alterations

**Figure 1:**
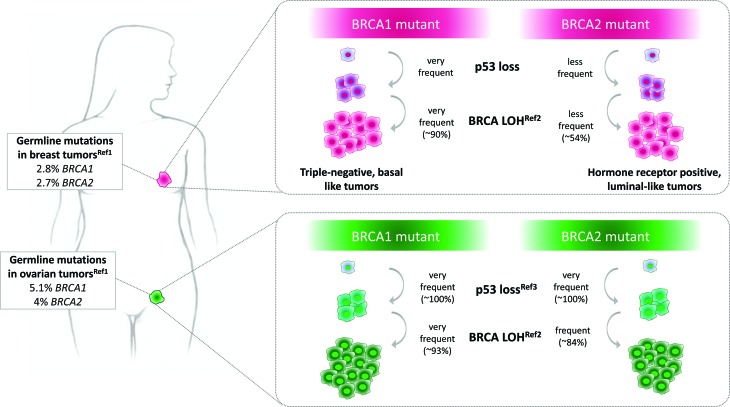
Frequent alterations arising in breast and ovarian tumors from patients carrying germline mutations in *BRCA1* and *BRCA2*. For details, see Ref 1 (Kurian *et al.*, 2017), Ref 2 (Maxwell *et al.*, 2017) and Ref3 (Network, 2011).

Initial whole-exome sequencing analyses of BRCA-associated breast and ovarian cancers have demonstrated, in a small number of tumors, that at base pair resolution the repertoire of somatic mutations that these cancers harbor is diverse (Figure 1) (Network, 2011, 2012). The most frequently mutated gene in both BRCA1 and BRCA2 tumors (breast and ovarian) is *TP53*. In addition, analysis of copy number alterations (CNAs) revealed that approximately 30% of these tumors harbored recurrent amplifications of *MYC* and *TERC.* For PALB2-BCs, the repertoire of somatic mutations is currently unknown.

A noteworthy genetic alteration observed in BRCA-associated tumors is the high frequency of somatic mutations affecting *TP53* (Crook *et al.*, 1997; Network 2011, 2012). The p53 protein, encoded by the *TP53* gene, is a potent transcription factor involved in many tumor suppressing mechanisms, such as cell cycle arrest, DNA repair, senescence and apoptosis (Vousden and Prives, 2009). Somatic *TP53* mutations have been reported in more than 60% of BRCA1-BCs but in lower frequency in BRCA2 breast tumors (Crook *et al.*, 1998; Manié *et al.*, 2009; Network, 2012). Interestingly, a significant proportion of *TP53* somatic mutations are protein-truncating (nonsense and frameshift mutations), suggesting strong selection for p53 loss-of-function rather than missense hotspot mutations (Holstege *et al.*, 2009). Also, a high prevalence of *TP53* mutations has also been observed in BRCA-associated ovarian cancers (Network 2011). In fact, the contribution of p53 to tumorigenesis of Brca tumors has been demonstrated in mouse models. *Brca1* ^+/-^Trp53^+/-^and *Brca2* ^+/-^Trp53^+/-^ mice show a slight increase in mammary carcinoma incidence compared with *Trp53* ^+/-^ mice (Cressman *et al.*, 1999; Jonkers *et al.*, 2001). As shown recently, in BCs, *TP53* mutations seem to be the second most common first event (after PTEN loss and *BRCA1* wild-type LOH) (Martins *et al.*, 2012). In ovarian cancer, *TP53* mutations seems to be a prerequisite to *BRCA1*-associated carcinogenesis, occurring before loss of the wild-type allele (Norquist *et al.*, 2010).

In addition to *TP53*, *PTEN* (phosphatase and tensin homolog) has also been shown to contribute to carcinogenesis of BRCA1-associated BC (Martins *et al.*, 2012). The protein product of *PTEN* is a potent inhibitor of the phosphatidylinositol 3-Kinase (PI3K) pathway, an oncogenic signaling cascade that promotes many of the cancer hallmarks (Carracedo and Pandolfi, 2008). Findings of *in vivo* studies have shown that mice carrying heterozygous inactivation of *PTEN* develop basal-like mammary tumors (Saal *et al.*, 2008). Additionally, in breast tumors arising in *BRCA1* mutations carriers, PTEN loss has been detected in more than 80% of the cases (Saal *et al.*, 2008; Phuah *et al.*, 2012). The inactivation of PTEN seems to contribute to the high rate of gene rearrangements involving DNA DSBs, intragenic inversions, insertions, and homozygous deletions found in BRCA1 tumors (Saal *et al.*, 2008). Moreover, in BRCA1 breast tumors, loss of PTEN has been show to precede *BRCA1* LOH and *TP53* mutation (Martins *et al.*, 2012). Interestingly, PTEN deficiency may also result in increased chromosomal instability due to its role in controlling the expression of RAD51 and cell cycle checkpoint (Shen *et al.*, 2007; Gupta *et al.*, 2009).

As mentioned previously, a common genetic alteration of BRCA1 and BRCA2 tumors is LOH. Although different studies have shown that most of BRCA tumors share this feature, findings demonstrating that *BRCA* wild-type allele may be preserved in a subset of cancer cells and that some BRCA tumors may not display loss of *BRCA* wild-type allele at all have raised issues regarding the true impact of the *BRCA* LOH on tumorigenesis (Osorio *et al.*, 2002; Tung *et al.*, 2010; Stefansson *et al.*, 2011; Martins *et al.*, 2012; Maxwell *et al.*, 2017). Several studies have found that in a substantial proportion of the cases, loss of the *BRCA* wild-type allele is not an initial event (Stefansson *et al.*, 2011; Martins *et al.*, 2012;). The findings obtained by Stefansson *et al.* (2011) support the hypothesis that loss of the *BRCA2* wild-type allele is a late, rather than early, event in progression of the disease. King *et al.* (2007) have suggested that LOH is not required for the tumorigenesis of BRCA breast tumors, since a high level of heterogeneity to this molecular event within and between pre-invasive lesions and invasive cancers was found. For PALB2-related BCs, the few reports to date have found controversial results regarding LOH of *PALB2* (Tischkowitz *et al.*, 2007; Hartley *et al.*, 2014).

It has also been found that BRCA-related tumors are characterized by a distinct mutational signature (signature 3), in which large deletions with overlapping microhomology at breakpoint junctions are found, likely associated with absence of BRCA1 or BRCA2 functions (Alexandrov *et al.*, 2013; Nik-Zainal *et al.*, 2016). Recently, it was demonstrated that, in contrast to tumors with biallelic germline inactivation of BRCA, single functional copies of BRCA (generally sufficient to maintain normal HR function) were not associated with signature 3 (Polak *et al.*, 2017).

With regard to CNAs, BRCA1 and BRCA2 breast tumors show different patterns of gains and losses compared to sporadic tumors (Jönsson *et al.*, 2005), and despite overlaps between BRCA1 and BRCA2 tumors many differences have been observed at this genomic level (van der Groep *et al.*, 2011). For PALB2 breast tumors, 1q gain, 20q gain, and 18q loss were consistently observed across tumors (Tischkowitz *et al.*, 2007). In BRCA-related epithelial ovarian carcinomas the few number of studies have yielded contradictory results. Despite the fact that some data indicate that somatic alterations do not differ substantially from the ones occurring in sporadic carcinomas (Kamieniak *et al.*, 2013), several reports have shown that BRCA ovarian cancers exhibit a significantly higher number of chromosomal aberrations and genomic imbalances than sporadic tumors (Israeli *et al.*, 2003; Walsh *et al.*, 2008).

## New therapeutic approaches

### Targeting homologous recombination deficiency

Many of the therapies newly developed for patients with *BRCA1* and *BRCA2*-mutated BCs explore the fact that these tumors lack DSB DNA repair by HR (Livraghi and Garber, 2015). The most promising therapies within this category are the inhibitors of poly (ADP-ribose) polymerase (PARP) (Evans and Matulonis, 2017). The discovery of the synthetic lethality interactions between PARP inhibitors and HR repair deficiency provided the basis for the clinical approval of olaparib in ovarian cancer and ongoing clinical trials of other drugs.

The PARPs are a large family of enzymes, which, in addition to other functions, participate in single-strand breaks (SSBs) repair via the base-excision repair (BER) pathway (Ashworth, 2008). Despite the importance of their role in the cellular DNA damage response, Parp1^-/-^ mice are viable, fertile and do not develop early onset tumors (Wang *et al.*, 1995; Conde *et al.*, 2001). However, the inability of Parp1^-/-^ cells repairing SSBs via PARP activity lead to stalling and collapse of replication forks in proliferation cells, transforming SSBs in DSBs, which may potentially be repaired by HR (Peng and Lin, 2011).

In 2005, two simultaneous publications demonstrated the impact of PARP inhibition in BRCA1 and BRCA2-deficient cells. The results of both studies showed that the complete dysfunction of BRCA proteins linked to PARP1 inhibition lead to chromosomal instability, cell cycle arrest, and apoptosis (Bryant *et al.*, 2005; Farmer *et al.*, 2005). These findings illustrate the concept of `synthetic lethality’, a phenomenon that occurs when the combination of two different mutations or cellular pathways inhibition lead to cell death, whereas one of the two events alone does not (Lord and Ashworth, 2017)

After *in vitro* and *in vivo* studies proved the synthetic lethality between PARP1 inhibition and *BRCA* dysfunction, an obvious next step was the validation of this paradigm in a clinical setting. Since then, several clinical trials have been launched to test the activity of different PARP inhibitors in the patient’s population carrying *BRCA* germline mutations. Several PARP inhibitors, including olaparib, niraparib, rucaparib, and BMN-673 are in different clinical phases of testing and have shown promising therapeutic activity such as in monotherapy (Fong *et al.*, 2009; Drew *et al.*, 2016; de Bono *et al.*, 2017).

The first-in-human phase I study of olaparib (also known as AZD2281) observed antitumor activity in breast and ovarian tumors arising in *BRCA* carriers, but not in patients without such mutations. In addition, minimal toxic effects, which are commonly associated with conventional chemotherapy, were observed. (Fong *et al.*, 2009). Subsequently, a phase II proof-of-concept trial provided evidences for the efficacy and tolerability of olaparib therapy in women carrying *BRCA* mutation and advanced-stage breast cancer (Tutt *et al.*, 2010). Similar results were obtained in an independent study including women with confirmed *BRCA* germline mutations and ovarian cancer (Audeh *et al.*, 2010). In 2015 a multicenter open-label phase II study including 298 BRCA mutation carriers which were refractory to standard therapy showed clinical benefit of olaparib in prostate and pancreatic cancer and confirmed activity in ovarian and breast cancer (Kaufman *et al.*, 2015). In 2014, olaparib was the first PARP inhibitor to receive regulatory approval in the United States and Europe to treat recurrent ovarian cancers associated to *BRCA* mutations as maintenance therapy postplatinum treatment. The accelerated approval was based on the results of the phase III SOLO2 study (Pujade-Lauraine *et al.*, 2017).

Initially found to induce synthetic lethality in preclinical model of BRCA loss-of-function (Jones *et al.*, 2009a), the first phase I study of niraparib (MK-4827), a highly selective inhibitor of PARP1 and PARP2, showed antitumor activity and a low frequency of high-grade toxic effects (Sandhu *et al.*, 2013). Subsequently, in a randomized, placebo-controlled, phase III trial it was demonstrated the efficacy and safety of niraparib as maintenance treatment in a broad population of patients with platinum-sensitive, recurrent ovarian cancer, regardless of the presence or absence of *BRCA1*, *BRCA2* mutations or HR deficiency status (Mirza *et al.*, 2016). This study was the basis for approval of the drug by the United States’ FDA in October 2016.

Talazoparib, another compound belonging to the PARP inhibitors class, initially showed encouraging clinical results. First tested *in vitro*, the drug selectively targeted tumor cells with *BRCA1*, *BRCA2*, or *PTEN* gene alterations with 20- to more than 200-fold greater potency than existing PARP1/2 inhibitors (such as olaparib, rucaparib, and veliparib) (Shen *et al.*, 2013). Preclinical results demonstrated that the potency in trapping PARP differed markedly among PARP inhibitors, a pattern not correlated with the catalytic inhibitory properties for each drug. However, preclinical potency may not necessarily translate into clinical efficacy, as other factors such as drug-related toxicities limiting dose escalation and patient selection come into play (Brown *et al.*, 2016).

In a pre-clinical study, rucaparib was found to be cytotoxic to *BRCA* mutated cells and associated with a reduction in growth of xenograft tumors harboring *BRCA* mutations (Drew *et al.*, 2011). In a phase II trial with BRCA-ovarian cancers, rucaparib was well tolerated and associated with stable disease (Drew *et al.*, 2016). Rucaparib was tested in two main clinical trials, ARIEL2 and ARIEL3. Data showed progression-free survival advantage for patients with BRCA mutant platinum-sensitive ovarian carcinomas. The drug was recently approved by the FDA (Swisher et al., 2017).

Over the past decade a new concept termed ‘BRCAness’ has been proposed. BRCAness was described as a phenomenon in which HR deficiency occurs in a tumor not due to a *BRCA1* or *BRCA2* germline mutation, but by mutations in other genes involved in HR (Lord and Ashworth, 2016). The experience with PARP inhibitors demonstrates that the use of this therapeutic approach may be expanded, including to other tumors with HR deficiency, regardless of tumor site (Riaz *et al.*, 2017). However, the clinical utility of this approach requires further validation (Frey and Pothuri, 2017).

More recently, some authors have suggested that patients with *BRCA1* or *BRCA2* germline mutations harbour a greater number of clonal mutations compared with *BRCA* wild-type tumors (Nik-Zainal *et al.*, 2012). This can lead to a more pronounced immunogenic phenotype and better response to immune checkpoint inhibitors (Dai *et al.*, 2018).

## Resistance mechanisms

Although PARP inhibitors have emerged as promising new therapeutic approaches for tumors arising in *BRCA* mutation carriers, drug resistance has become an important clinical issue. The investigation of the multiple potential resistance mechanisms has led to the identification of both processing operating through the drug target and under *BRCA1*, *BRCA2,* and their pathways (Lord and Ashworth, 2013).

Discovered independently by two groups, secondary mutation is the most common mechanism of acquired resistance to PARP inhibitors. Edwards *et al.* (2008) using the CAPAN1 pancreatic cancer cell line which harbors a *BRCA2* frameshift mutation (c.6174delT), found that resistant clones to PARP inhibitors could form RAD51 nuclear foci and prevent genomic instability, both of which are hallmarks of an efficient HR. These resistant clones displayed a secondary *BRCA2* intragenic deletion of the region containing c.6174delT mutation and restoration of the open reading frame (ORF), resulting in the expression of new BRCA2 isoforms (Edwards *et al.*, 2008). Similar results were also observed in cisplatin-resistant *BRCA2*-mutated breast-cancer cell line (Sakai *et al.*, 2008). In ovarian cancers, secondary mutations restoring the *BRCA2* ORF were also observed in patients who become resistant to platinum salts (Edwards *et al.*, 2008; Sakai *et al.*, 2008). Barber *et al.* (2013) analyzed resistance to olaparib in a male patient with BC and a woman with breast and ovarian cancer that were enrolled in a phase II clinical trial. Both were carriers of a truncating *BRCA2* mutation and presented multiple metastatic lesions. Deep sequencing of treatment-naive and olaparib-resistant lesions from both patients indicated the emergence of secondary mutations that potentially restored de ORF of *BRCA2* gene only in the resistant lesions (Barber *et al.*, 2013). Taken together, these data provide evidence that, at least in a subset of patients, platinum salts and PARP inhibitors require defective HR for their antitumor activity. Although the frequency of secondary *BRCA* mutations is not precisely known, this is the most well validated mechanism of resistance to PARP inhibitors in the population of patients carrying *BRCA* mutations.

Reduced activity of p53 binding protein 1 (53BP1) has also been suggested to be a potential resistance mechanism to PARP inhibitors (Lord and Ashworth, 2013). Initial studies have showed that mouse embryonic fibroblasts without a full-length form of BRCA1 and deleted 53bp1 are defective in induction of senescence and cell death. Furthermore, *in vivo* results confirmed that the embryonic lethality associated with complete BRCA1-deficiency may be alleviated by 53bp1 deletion (Cao *et al.*, 2009). Bouwman *et al.* (2010) showed that loss of 53BP1 partially restores the HR defect of BRCA1-deficient cells and reverts their hypersensitivity to DNA-damaging agents. Moreover, these findings have potential clinical implications, given that reduced 53BP1 expression was found in a subset of sporadic triple-negative and BRCA-associated BCs (Bouwman *et al.*, 2010). Further study in a mouse model of Brca1 deficiency showed that mammary gland tumors that initially were sensitive to olaparib developed resistance associated with 53bp1 factor. In a subset of the cases (3 out of 11), this resistance was caused by partial restoration of HR due to somatic loss of 53BP1 (Jaspers *et al.*, 2013). On the other hand, 53bp1 depletion did not have any effect on cells with BRCA2 deficiency.

## Conclusion and perspectives

After two decades of efforts, we have witnessed remarkable advances in our understanding of basic aspects of *BRCA* and *PALB2* genes. The roles of these genes in DNA repair by HR and the discovery of synthetic lethal interaction between PARP inhibition and BRCA1 or BRCA2 deficiency allowed us to make significant progress in the clinical setting. However, many questions remain. For example, although the identification of abnormal phenotypes has been described even in normal cells of *BRCA* and *PALB2* germline mutation carriers, suggesting haploinsufficiency for specific BRCA functions, the contribution of this finding to cancer predisposition still remains controversial. Additionally, the molecular basis underlying the tissue-specificity of cancer predisposition associated with germline *BRCA* and *PALB2* mutations as well as the impact of the *BRCA* or *PALB2* wild-type allele (absence of LOH) within tumors on the DNA repair by HR and response to therapies requires further evaluation. Finally, our complete understanding of the molecular abnormalities in BRCA and PALB2-associated tumors will not only provide insights into the pathogenesis of these cancers, but also will help to identify novel targets for therapies as well as predictive markers for HR deficiency and drug response.
